# Distinct muscarinic acetylcholine receptor subtypes mediate pre- and postsynaptic effects in rat neocortex

**DOI:** 10.1186/1471-2202-13-42

**Published:** 2012-04-27

**Authors:** Sylvain Gigout, Gareth A Jones, Stephan Wierschke, Ceri H Davies, Jeannette M Watson, Rudolf A Deisz

**Affiliations:** 1 Center for Anatomy, Institute for Cell Biology and Neurobiology, Charité Universitätsmedizin Berlin, Philippstr. 12, 10115, Berlin, Germany; 2 GlaxoSmithKline, Centre of Excellence for Drug Discovery, Third Avenue, Harlow, Essex, CM19 5AW, UK

**Keywords:** Carbachol, M-current, Muscarinic acetylcholine receptors, Sensorimotor cortex, Cognition, Synaptic transmission

## Abstract

**Background:**

Cholinergic transmission has been implicated in learning, memory and cognition. However, the cellular effects induced by muscarinic acetylcholine receptors (mAChRs) activation are poorly understood in the neocortex. We investigated the effects of the cholinergic agonist carbachol (CCh) and various agonists and antagonists on neuronal activity in rat neocortical slices using intracellular (sharp microelectrode) and field potential recordings.

**Results:**

CCh increased neuronal firing but reduced synaptic transmission. The increase of neuronal firing was antagonized by pirenzepine (M_1_/M_4_ mAChRs antagonist) but not by AF-DX 116 (M_2_/M_4_ mAChRs antagonist). Pirenzepine reversed the depressant effect of CCh on excitatory postsynaptic potential (EPSP) but had marginal effects when applied before CCh. AF-DX 116 antagonized the depression of EPSP when applied before or during CCh. CCh also decreased the paired-pulse inhibition of field potentials and the inhibitory conductances mediated by GABA_A_ and GABA_B_ receptors. The depression of paired-pulse inhibition was antagonized or prevented by AF-DX 116 or atropine but only marginally by pirenzepine. The inhibitory conductances were unaltered by xanomeline (M_1_/M_4_ mAChRs agonist), yet the CCh-induced depression was antagonized by AF-DX 116. Linopirdine, a selective M-current blocker, mimicked the effect of CCh on neuronal firing. However, linopirdine had no effect on the amplitude of EPSP or on the paired-pulse inhibition, indicating that M-current is involved in the increase of neuronal excitability but neither in the depression of EPSP nor paired-pulse inhibition.

**Conclusions:**

These data indicate that the three effects are mediated by different mAChRs, the increase in firing being mediated by M_1_ mAChR, decrease of inhibition by M_2_ mAChR and depression of excitatory transmission by M_4_ mAChR. The depression of EPSP and increase of neuronal firing might enhance the signal-to-noise ratio, whereas the concomitant depression of inhibition would facilitate long-term potentiation. Thus, this triade of effects may represent a “neuronal correlate” of attention and learning.

## Background

Considerable evidence indicates a crucial role of acetylcholine (ACh)-mediated transmission in learning and memory. Impairment of cholinergic signalling contributes to cognitive deficits in animal models [1] and diseases such as schizophrenia and Alzheimer’s Disease [2-4]. Conversely, drugs that augment cholinergic transmission (e.g. donepezil or galatamine) have been shown to improve certain cognitive functions [5,6]. In this context metabotropic ACh receptors, termed muscarinic ACh receptors (mAChRs) [7] play a crucial role. Five mAChR subtypes have been cloned (M_1_-M_5_); each consisting of seven highly conserved transmembranal segments. A large and more variable intracellular domain [8] couples the receptor to second messengers and ion channels via heterotrimeric G-proteins [1,7,9,10].

The mAChRs are now recognized as key targets for the treatment of different neuropathologies such as Alzheimer’s Disease, schizophrenia and Parkinson’s Disease [10]. In particular, agonists of M_1_ mAChR or antagonists at M_2_ or M_4_ mAChRs are promising substances [10,11].

However, the similarity in ligand-binding sites between mAChR subtypes, the paucity of selective agonists/antagonists as well as the expression of different subtypes of mAChRs in a given area constitute major obstacles to delineate cellular effects of a given subtype [1]. The improvement of therapeutic approaches using specific mAChR modulators is further impeded by a lack of established and validated protocol to screen efficacy of different mAChR agonist/antagonist at native receptors in neocortical slices. Characterization of receptors in a native system is a crucial issue. For instance, at D_2_-type dopamine receptors [12] or AMPA receptors [13], the pharmacology at recombinant receptors critically depends on the experimental conditions. Thus, the pharmacology of mAChR in heterologous systems may not necessarily relate to the natively expressed mAChR [14]. Therefore, to delineate the pharmacology of mAChRs in the neocortex, the data must be obtained in the native neocortical slice.

We tried to define simple paradigms to quantify routinely the efficacy of different mAChR agonists. This should facilitate further screening for novel drugs in the neocortex. We investigated the effects of CCh and various mAChR antagonists on several parameters of neuronal excitability and the synaptic transmission in neocortical layer II/III. We have chosen layer II/III of neocortex because of the established expression of M_1_, M_2_ and M_4_ but not M_3_ and M_5_ mAChRs at the mRNA [15] and the protein levels [15-18].

As a first step we tested whether the published effects obtained under various experimental conditions can be reproduced. Firstly, in neocortical pyramidal neurones, ACh or the prototype cholinergic agonist carbamylcholine-chloride (carbachol, CCh) depolarizes the membrane potential and increases action potential (AP) firing [19-21]. Secondly, CCh reduces excitatory postsynaptic potentials (EPSPs) in neocortical neurons [21,22]. This depressant effect was reduced by the preferential M_1_/M_4_ and M_2_/M_4_ mAChRs antagonists (pirenzepine and AFDX 116, respectively) and was not due to activation of nicotinic receptors [22]. Thirdly, ACh reduces the GABA release in neocortex via activation of M_2_ mAChR and to a smaller extent by activation of M_1_ mAChR [23].

Here we show that these 3 effects are mediated by 3 different subtypes of mAChRs allowing screening for mAChRs subtype-specific agonists/antagonists.

## Results

### Modulation of neuronal properties and firing behaviour

In control conditions, neurones had a resting membrane potential (E_m_) of −76.2 ± 0.4 mV, an input resistance of 20.4 ± 0.8 MΩ and the AP amplitudes averaged 92.4 ± 0.7 mV (n = 92). The slope of neuronal AP firing was 57.3 ± 1.6 APs/nA in control conditions.

#### Effects of CCh on membrane properties and neuronal firing

**Figure 1:**
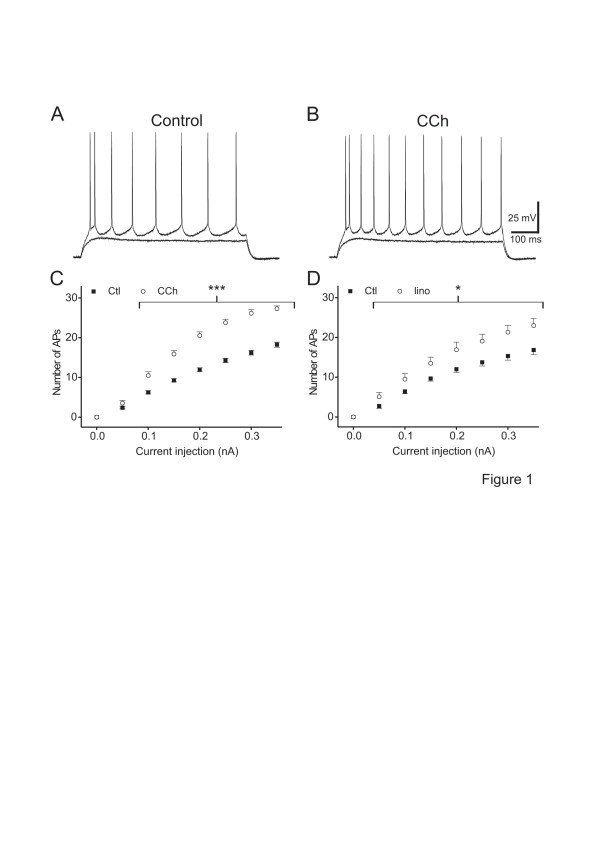
**CCh and linopirdine effects on neuronal AP firing.** A, B. Voltage traces of a neocortical neurone in control condition (A) and in the presence of 10 μM CCh (B). The traces show voltage responses to current injections of 0.40 and 0.50 nA (durations 600 ms). Note the increased number of APs in the presence of 10 μM CCh compared to control condition. C, D. Plot of the average number of APs *vs.* current injection in control conditions and in the presence of 10 μM CCh (C; n = 40) or 10 μM linopirdine (D, n = 12). These experiments were performed in STRC. *: *p* < 0.05 *vs.* control, ***: *p* < 0.001 *vs.* control

**Figure 2:**
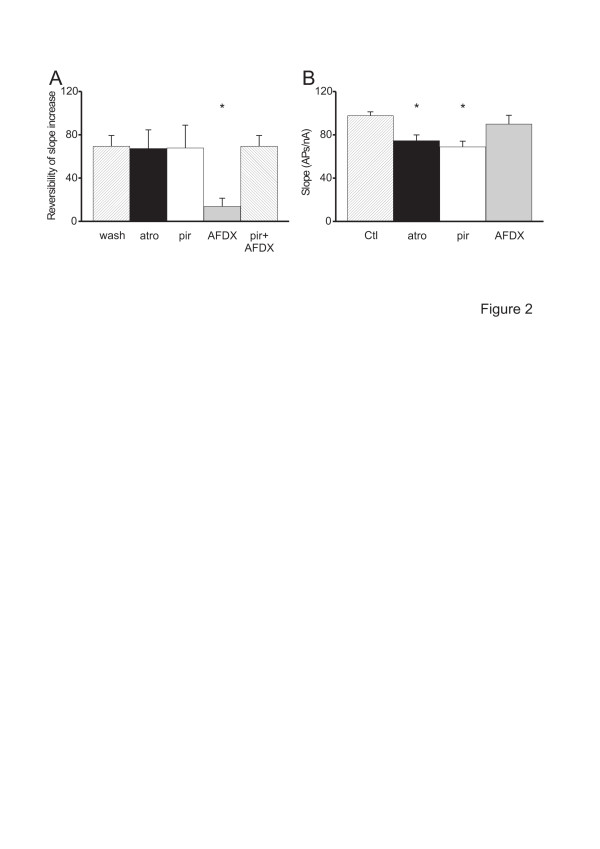
**Pharmacology of CCh-induced increase in slope of neuronal APs firing.** A. Plot of the reversibility (in %) of the CCh-induced increase in slope of neuronal APs firing after washout or addition of different mAChR antagonists to a CCh-containing ACSF as indicated. Note the reversibility observed with atropine (n = 7) or pirenzepine (n = 7) was maximal since not different to that observed after washout (n = 10). However, the reversibility observed with AFDX was much smaller to that observed after washout. The reversibility observed during a co-application of CCh with “AFDX + pirenzepine” (n = 8) was not different to that obtained during a co-application of CCh with “pirenzepine alone” (n = 7). These experiments were performed in STRC. *: *p* < 0.05 *vs.* reversibility after washout. B. Plot of the slope of neuronal AP firing during CCh addition in standard ACSF or in ACSF containing different mAChR antagonists as indicated. Note the slope of neuronal firing during application of CCh in a atropine-containing (n = 9) or a pirenzepine-containing (n = 7) ACSF is significantly smaller to that obtained during application of CCh in standard ACSF (n = 40). However, slope of neuronal firing during application of CCh in an AFDX-containing ACSF (n = 7) is not different to that obtained during application of CCh in standard ACSF (n = 40). These experiments were performed in STRC. The symbols represent: *: *p* < 0.05 *vs.* slope of neuronal firing during application of CCh in standard ACSF

Bath application of CCh had no effect on E_m_ (control: -75.5 ± 0.7 mV, CCh: -74.8 ± 0.7 mV; n = 40, *p* > 0.05) or AP amplitudes (control: 89.5 ± 1.2 mV, CCh: 88.5 ± 1.3 mV; n = 40, *p* > 0.05). However, CCh consistently increased the neuronal input resistance (control: 20.4 ± 1.3 MΩ, CCh: 24.2 ± 1.4 MΩ; n = 40, *p* < 0.002). During CCh application a given current injection elicited more APs (Figure 1A, B, C). The slope of neuronal firing averaged 58.4 ± 2.1 APs/nA in control and increased to 97.8 ± 3.4 APs/nA in the presence of CCh (n = 40, *p* < 0.0001), much more than anticipated from the increase in membrane resistance. The reversibility of CCh effect on slope of neuronal APs firing was by 66.4 ± 6.0% after washout (n = 10; Figure 2A). The sub-rheobase for AP generation was significantly decreased during perfusion with CCh (control: 0.5 ± 0.02 nA, CCh: 0.46 ± 0.02 nA, n = 40, *p* < 0.01).

#### Pharmacological delineation of the mAChR subtypes involved

Atropine (n = 7) or pirenzepine (n = 7) reversed the CCh-induced increase of slope of neuronal firing (*p* < 0.05 *vs* CCh alone and *p* > 0.05 *vs.* control; not shown). This antagonism was almost complete and indistinguishable from the reversibility observed after washout (n = 10; *p* > 0.05; Figure 2A). However, AFDX did not reverse the CCh-induced increase of slope of neuronal firing (*p* > 0.05 *vs.* CCh and *p* < 0.05 *vs.* control). In fact, the antagonism observed with AFDX (n = 7) was much smaller than the reversibility observed after washout (n = 10, *p* < 0.01; Figure 2A). In the continuous presence of CCh, the antagonism observed during a co-application of “pirenzepine + AFDX” (n = 8) was not different to that obtained during application of pirenzepine alone (n = 7, *p* > 0.05; Figure 2A). This suggests that the slight antagonism observed during application of AFDX alone is due to some antagonistic effect of AFDX on M_1_ mAChR.

The slope of neuronal firing during application of CCh in atropine-containing (n = 9) or pirenzepine-containing artificial cerebrospinal fluid (ACSF) (n = 7) is significantly smaller than that obtained during application of CCh in standard ACSF (n = 40, *p* < 0.01; Figure 2B). However, the slope of neuronal firing during application of CCh added to an AFDX-containing ACSF (n = 7) is not different to that obtained during application of CCh in standard ACSF (n = 40, *p* > 0.05; Figure 2B), i.e. AFDX fails to modify the CCh-induced effects on firing. To further test the involvement of M_1_ mAChR in the effects on firing, we applied xanomeline, a M_1_/M_4_ preferring mAChRs agonist [24]. Xanomeline increased the slope of neuronal firing from 57.8 ± 6.5 APs/nA to 80.4 ± 7.7 APs/nA (n = 11, *p* < 0.001).

Together these data suggest that CCh exerts its action on slope of neuronal firing via mAChRs, and in particular M_1_ but not M_2_ or M_4_ mAChRs.

#### CCh-induced increase of neuronal firing is mimicked by M-current blockade

To test the involvement of K_V_7 channels blockade [9] in the M_1_ mAChR-mediated increase of neuronal firing, we applied linopirdine (10 μM), a blocker of K_V_7 channels [25]. Bath application of linopirdine had no effect on AP amplitudes (control: 97.5 ± 1.6 mV, linopirdine: 95.4 ± 2.5 mV; n = 12, *p* > 0.05) or on E_m_ (control: -76.5 ± 1.1 mV, linopirdine: -75.6 ± 1.2 mV; n = 12, *p* > 0.05) as expected since K_V_7 channels are not activated at −75 mV [9]. However, linopirdine increased the neuronal input resistance (control: 17.8 ± 1.8 MΩ, linopirdine: 28.4 ± 3.2 MΩ; n = 12; *p* < 0.0001).

Linopirdine increased significantly the number of APs at each current magnitude (Figure 1D). Consequently, the slope was 58.4 ± 5.1 APs/nA in control and increased to 79.8 ± 7.8 APs/nA in the presence of linopirdine (n = 12, *p* < 0.002). The sub-rheobase for AP generation was strongly decreased from 0.58 ± 0.05 nA in control to 0.36 ± 0.03 nA in the presence of linopirdine (n = 12, *p* < 0.0001).

Bath application of retigabine (10 μM), an activator of K_V_7 channels [26], had no effect on AP amplitudes (control: 95.6 ± 1.6 mV, retigabine: 95.1 ± 2.8 mV; n = 7, *p* > 0.05), on E_m_ (control: -81.5 ± 1.6 mV, retigabine: -81.9 ± 1.6 mV; n = 7, *p* > 0.05), or on neuronal input resistance (control: 15.3 ± 3.3 MΩ, retigabine: 13.3 ± 1.8 MΩ; n = 7; *p* > 0.05). Retigabine decreased significantly the number of APs at each current magnitude (not shown). Consequently, the slope was 53.9 ± 6.1 APs/nA in control and decreased to 12.2 ± 1.9 APs/nA in the presence of retigabine (n = 7, *p* < 0.0002). The sub-rheobase for AP generation was increased from 0.81 ± 0.09 nA in control to 0.99 ± 0.12 nA in the presence of retigabine (n = 7, *p* < 0.01).

### Modulation of GABAergic transmission

#### CCh decreases the paired-pulse inhibition of field potentials

Paired-pulse protocols are often used to study GABAergic inhibition in field potentials [27]. We therefore employed a paired-pulse protocol as a first step towards studying CCh modulation of inhibition.

The input–output curves obtained in ITRC (n = 49; not shown) were qualitatively similar to that obtained in STRC, except for larger amplitudes of potentials (*p* < 0.0001), as expected [28]. The amplitude of the field potentials decreased significantly in the presence of CCh (10 μM) at stimulus intensities larger than 0.3 mA (not shown; n = 49, *p* < 0.05).

This CCh-induced synaptic depression was antagonized by atropine (n = 19). Pirenzepine (n = 15) and AFDX (n = 16) at least partially reversed the CCh-induced depression. Linopirdine (n = 10, *p* > 0.05) and retigabine (n = 8, *p* > 0.05) had no effect on synaptic transmission (data not shown).

Paired-pulse stimulations (interpulse intervals: 10 ms - 1 s) were used to determine the magnitude of paired-pulse depression of the second field potentials (FP2). Since the apparent time-to-peak of the intracellular GABA_A_-mediated response is about 20–30 ms [29], we focus here on the results obtained for interpulse intervals of 10–30 ms to investigate GABA_A_ receptor-mediated inhibition. A high stimulus intensity (1 mA) was used to maximally activate GABAergic transmission. When two pulses were delivered 20 ms apart, the mean amplitude of the FP2 was 25 ± 4% of the amplitude of the first (FP1; n = 59).

**Figure 3:**
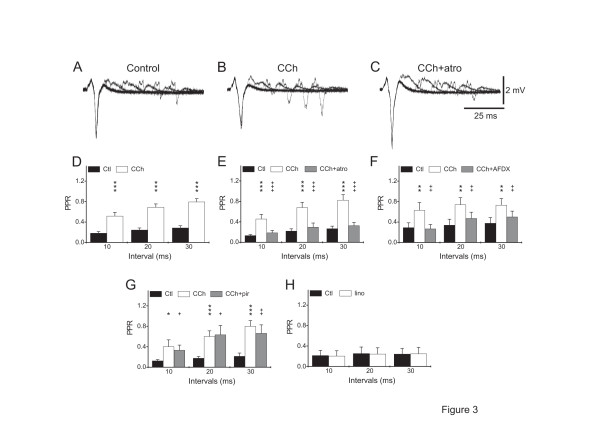
**Pharmacology of CCh effect on paired-pulse stimulation of field potentials.** A, B, C. Example of field potential recordings using a paired-pulse protocol in control (A), 10 μM CCh (B) and during 10 μM CCh + 1 μM atropine co-application (C). Note that 10 μM CCh decreased the paired pulse depression compared to control condition. Co-application of 1 μM atropine with 10 μM CCh reversed this effect. D. Plots of the paired-pulse ratio (PPR) at different interstimulus intervals (10, 20 and 30 ms), in control and in the presence of 10 μM CCh (n = 49). E - H. The effects of different mAChR antagonists and of 10 μM linopirdine on CCh-induced alterations of paired-pulse ratio, as indicated. Note that co-application of 1 μM atropine (E; n = 19) or 2 μM AFDX (F; n = 15) with CCh reversed the CCh-mediated increase of PPR, whereas 1 μM pirenzepine (G; n = 14) had little effects. Note that linopirdine (H; n = 10) did not mimick the CCh-induced increase of PPR. These experiments were performed in an ITRC. The symbols represent: *: *p* < 0.05 *vs.* control, **: *p* < 0.01 *vs.* control, ***: *p* < 0.001 *vs.* control; +: *p* < 0.05 *vs.* CCh, ++: *p* < 0.01 *vs.* CCh, +++: *p* < 0.001 *vs.* CCh

CCh (10 μM) decreased slightly the amplitude of FP1 (to 92 ± 15% of control value; n = 49, *p* < 0.05; Figure 3A, B), whereas the amplitude of FP2 increased strongly (to 342 ± 50% of the control value; n = 49, *p* < 0.0001) for an interpulse interval of 20 ms (Figure 3A, B). This resulted in an increase of the paired-pulse ratio (PPR) which was significant for interstimulus intervals from 10 ms to 400 ms (control: 0.24 ± 0.04, CCh: 0.69 ± 0.06, interstimulus interval 20 ms; n = 49, *p* < 0.0001; Figure 3D).

### CCh decreases the paired-pulse inhibition via mAChRs

At interpulse intervals between 10 and 30 ms, both atropine (n = 19, *p* < 0.05 *vs.* CCh and *p* > 0.05 *vs.* control; Figure 3A, B, C, E) and AFDX (n = 15, *p* < 0.05 *vs.* CCh and *p* > 0.05 *vs.* control; Figure 3F) antagonized the CCh-induced increase in PPR. In contrast, the CCh-induced increase of the PPR at these interstimulus intervals was unaltered by addition of pirenzepine (n = 14, *p* > 0.05 *vs.* CCh and *p* < 0.05 *vs.* control; Figure 3G). Conversely, application of AFDX (n = 6) or atropine (n = 5) prevented the CCh-induced increase of PPR (*p* > 0.05 *vs.* control; not shown), whereas pirenzepine failed to prevent this CCh-induced increase of PPR (n = 7, *p* < 0.05 *vs.* control; not shown).

Application of linopirdine had no effect on the PPR (n = 10, *p* > 0.68; Figure 3H) suggesting that the M-current blockade is not involved in the CCh-induced increase of PPR. Additionally, we verified that retigabine (10 μM) did not alter the PPR (not shown, n = 8, *p* > 0.05). This suggests that modulation of GABA release, underlying the paired-pulse depression, is dominated by M_2_ mAChR effects. To further rule out the involvement of M_1_/M_4_ mAChRs in the depression of inhibition, we applied xanomeline. Xanomeline did not alter the PPR (n = 6, *p* > 0.05).

#### CCh decreases inhibitory synaptic conductances via M_2_ mAChR

**Figure 4:**
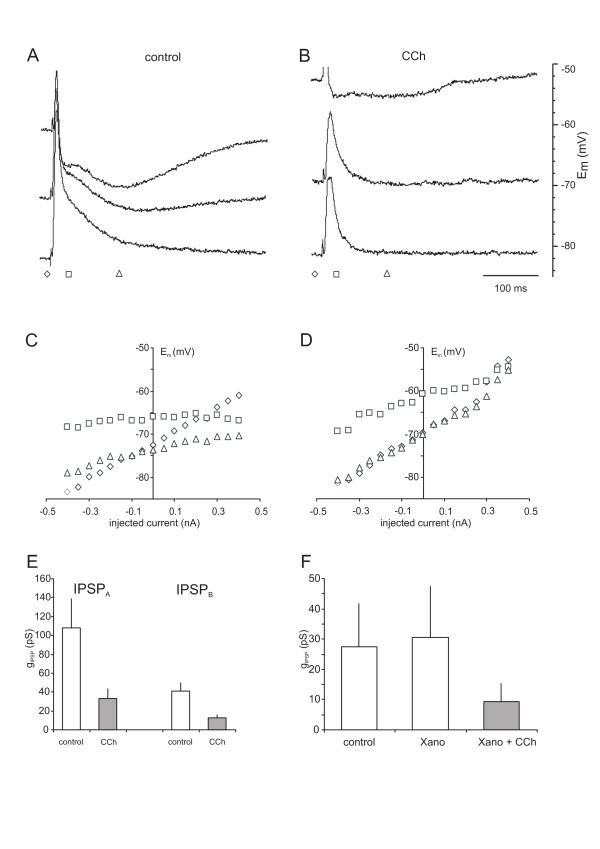
**Effects of cholinergic compounds on synaptic inhibition.** A, B. Voltage traces of a neurone in control conditions and in the presence of CCh. Orthodromic stimulation elicited compound synaptic responses consisting of an EPSP, IPSP_A_ and IPSP_B_. The two IPSPs are particularly obvious at less negative membrane potentials. The membrane potential was altered by current injections (+0.4, 0, -0.4 nA, from top to bottom). B: Traces from the same neurone in the presence of CCh. Note the marked decrease in the amplitudes of IPSP_A_ and IPSP_B_ in the top trace. C,D: Plot of the membrane potentials obtained by different current injections (−0.4 to + 0.4, 0.05 nA increment) at different times of the traces shown in A and B. The different symbols denote the different times indicated in A and B. Note the decrease in IPSP_A_ and IPSP_B_ conductances obvious from the steeper current voltage relationship. E. Plot of the mean g_IPSP-A_ and g_IPSP-B_ in control and in the presence of CCh as indicated. F. Plot of the mean g_IPSP-B_ values in control, in the presence of xanomeline (xano) and xanomeline + CCh. The vertical bars represent the s.e.m

To more directly evaluate the effects of mAChRs on synaptic inhibition, we evaluated the synaptic conductances underlying the IPSP_A_ and IPSP_B_ in the presence of various mAChR related compounds. Application of CCh greatly reduced the amplitudes of both, IPSP_A_ and IPSP_B_ components (Figure 4 A,B). Estimating the underlying conductances (g_IPSP_) revealed substantial decreases; g_IPSP-A_ decreased from 110 ± 31 nS to 34 ± 11 nS, g_IPSP-B_ decreased from 41 ± 9 nS to 12 ± 3 nS, i.e. by 69.1 and 69.8%, respectively (Figure 4E; n = 17, *p* < 0.05). To evaluate which subtypes of mAChRs are involved in this modulation of inhibition we first tested the M_2/_M_4_ mAChRs antagonist AFDX in the presence of CCh. In these neurones, CCh virtually abolished the conductances of both IPSPs (g_IPSP-A_ from 32.8 ± 13.6 to 0.0 ± 5.4 nS; n = 5, *p* < 0.05 and g_IPSP-B_ from 18.7 ± 4.6 nS to 3.5 ± 1.4 nS; n = 5, *p* < 0.05). Application of AFDX caused a considerable reversal in the depression of inhibition recovering to 63 and 72% of the control values (*p* > 0.05 *vs.* control).

Next we tested the effects of the M_1_/M_4_ mAChRs antagonist pirenzepine. Application of CCh considerably reduced the synaptic conductances, g_IPSP-A_ from 104.3 ± 39.4 nS to 58.9 ± 29.0 nS, g_IPSP-B_ from 25.0 ± 8.2 nS to 13.0 ± 5.3 nS i.e. on average by 44 and 48%. On addition of pirenzepine, the depression of inhibitory conductances was essentially unaltered (g_IPSP-A_: 74.9 nS, *p* > 0.05 *vs.* CCh; g_IPSP-B_: 15.7 nS *p* > 0.05 *vs.* CCh).

To further substantiate our hypothesis of a M_2_ mAChR-mediated depression of inhibition, we next tested xanomeline to evaluate possible effects of M_1_ or M_4_ mAChRs. Application of xanomeline (3 μM) had no consistent effect on E_m_ or R_m_ (control −75.2 ± 2.0 mV, 26.7 ± 9.0 MΩ xanomeline: -76.4 ± 1.7 mV, 26.1 ± 9.7 MΩ *p* > 0.05). These neurones exhibited the usual alteration of slope of firing (increasing from 64.4 ± 10.7 to 79.4 ± 10.9 APs/nA) and the depression of EPSP amplitudes (from 25.0 ± 1.7 to 23.8 ± 1.2). Yet, the g_IPSP-A_ and g_IPSP-B_ were unaltered (control: 88 ± 29 nS, 28 ± 7 nS, xanomeline: 92 ± 28 nS, 31 ± 8 nS, respectively, n = 5, *p* > 0.05, see Figure 4 F). Addition of 10 μM CCh in the presence of xanomeline had no further effect on E_m_ or R_m_ (*p* > 0.05 *vs.* CCh) but greatly reduced both inhibitory conductances, g_IPSP-A_ decreased to 36 ± 18 nS and g_IPSP-B_ to 9 ± 3 nS, i.e. by 61.2 and 69.1%, close to the values obtained for CCh alone. The difference to control was significant for g_IPSP-B_ (*p* < 0.05), but just not significant for the g_IPSP-A_ (*p* = 0.057).

### Modulation of glutamatergic transmission

#### Effects of carbachol on excitatory synaptic transmission: Field potentials and intracellular recordings

**Figure 5:**
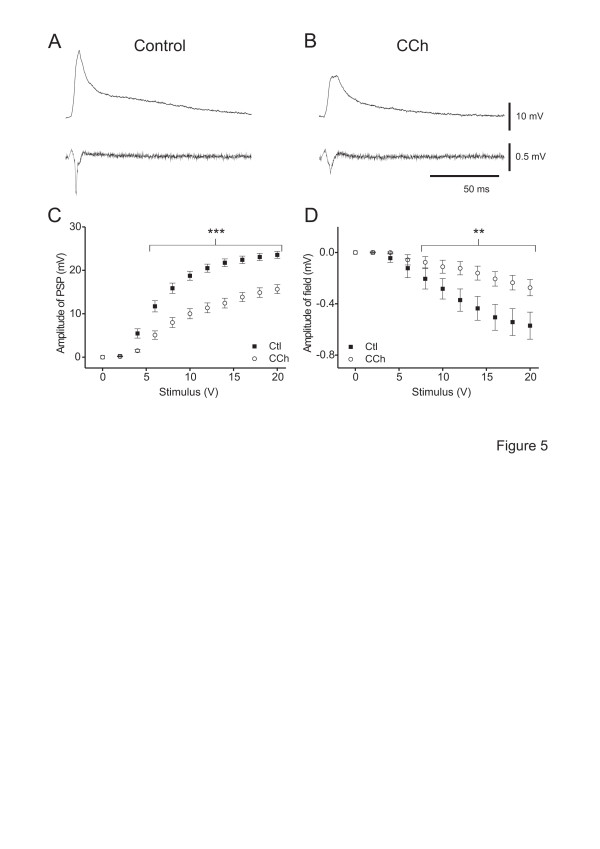
**CCh-induced depression of synaptic transmission is due to activation of muscarinic receptors.** A, B. Voltage traces of a rat neocortical neurone (top traces) and simultaneously recorded field potentials (bottom trace) in control condition (A) and in the presence of 10 μM carbachol (CCh) (B). The traces show synaptic responses elicited by orthodromic stimulation (100 μs, 20 V). Note the depressant effect of CCh on the amplitude of EPSP and field potential. C. Plot of the average EPSP amplitudes *vs.* stimulus intensity (input–output curve) in control conditions and in the presence of CCh as indicated (n = 45). D. Plot of the average field potential amplitudes *vs.* stimulus intensity in control and in the presence of CCh (n = 42). These experiments were performed in STRC. **: *p* < 0.01, ***: *p* < 0.001

In control ACSF, the amplitudes of EPSP and field potentials increased progressively with increasing stimulus intensities to reach a plateau (Figure 5C, D). Application of CCh (10 μM) decreased the amplitude of both EPSP (n = 45, *p* < 0.001; Figure 5A, B, C) and field potentials (STRC; n = 42, *p* < 0.05; Figure 5A, B, D) in a similar range of stimulus intensities.

The input–output curves of EPSP were well fitted by the Boltzmann equation yielding a maximal EPSP amplitude (EPSP_max_) of 22.6 ± 0.9 mV, a half maximal stimulus intensity (I_50_) of 6.71 ± 0.36 V and a slope factor of 1.66 ± 0.14 (n = 45). CCh significantly decreased EPSP_max_ to 16.0 ± 1.1 mV and increased both I_50_ and slope factor to 9.08 ± 0.44 V and 2.82 ± 0.24, respectively (n = 45; *p* < 0.001), i.e. the curve became more shallow and I_50_ shifted to higher values.

The depression of field potentials by CCh (n = 8) was completely reversible (*p* > 0.05 *vs.* control), whereas that of EPSPs (n = 11) was partially reversible after washout. The EPSP_max_ recovered on average by 66 ± 11% after washout (*p* < 0.05 *vs.* control and CCh), whereas the effects on I_50_ and slope factor were completely reversible (*p* > 0.05 *vs.* control, n = 11).

#### CCh-induced depression of synaptic responses is due to activation of mAChRs

Atropine (1 μM) alone had no effect on the amplitude of EPSP or field potentials at all stimulus intensities (2–20 V; n = 9, *p* > 0.05; not shown). Also, I_50_ or slope factor were unaffected by atropine (not shown), indicating no obvious steady state modulation of synaptic responses by endogeneous mAChRs. Pre-application of atropine prevented the CCh-induced decrease of EPSP_max_ as well as the CCh-induced increase of I_50_ and slope factor (n = 7, *p* > 0.05 *vs.* atropine or *vs.* control; Table 1). Conversely, addition of atropine fully reversed the CCh-induced decrease of EPSP_max_ and field potential amplitude (n = 7, *p* > 0.05 *vs.* control and *p* < 0.05 *vs.* CCh; Table 1). The CCh-induced increase of I_50_ and slope factor were also reversed (n = 7, *p* > 0.05 *vs.* control and *p* < 0.05 *vs.* CCh; Table 1). This suggests that the CCh effects on excitatory synaptic transmission are mediated by activation of mAChRs.

**Table 1:** **Effects of 10 μM CCh, 1 μM atropine alone, or co-application of CCh and atropine as indicated on the maximal amplitude of the field potentials, the maximal amplitude of EPSP (EPSP**_**max**_**), the I**_**50**_**and slope factor of the EPSP input–output curve**

	**Ctl (n = 7)**	**CCh (n = 7)**	**CCh + atro (n = 7)**
**Field max (mV)**	−0.53 ± 0.17	−0.16 ± 0.05 (*)	−0.61 ± 0.15 (+)
**EPSP max (mV)**	18.8 ± 1.6	11.8 ± 1.6 (*)	19.1 ± 2.2 (+)
**I**_**50**_**(V)**	7.25 ± 1.15	8.36 ± 1.11 (*)	6.39 ± 1.13 (+)
**Slope factor**	1.44 ± 0.41	2.75 ± 0.2 (*)	1.26 ± 0.43 (+)
	**Ctl (n = 7)**	**atro (n = 7)**	**atro + CCh (n = 7)**
**Field max (mV)**	−0.63 ± 0.16	−0.73 ± 0.2 (*)	−0.78 ± 0.21 (*)
**EPSP max (mV)**	21 ± 1.7	21 ± 1.8	21.8 ± 1.9
**I**_**50**_**(V)**	9.77 ± 0.78	9.5 ± 0.8	8.92 ± 0.62
**Slope factor**	2.12 ± 0.36	2.05 ± 0.29	2.26 ± 0.12

The order of two drugs given in the figures indicates the sequence of application (i.e. “CCh + atro” means atropine was added to a CCh containing ACSF and vice versa)

These experiments were performed in STRC

*: *p* < 0.05 *vs.* control, +: *p* < 0.05 *vs.* previous condition

#### The CCh-induced depression of synaptic responses is sensitive to pirenzepine and AFDX

Applied alone, neither pirenzepine (1 μM; n = 10) nor AFDX (2 μM; n = 11) had detectable effects on the EPSP amplitude, I_50_ or slope factor of the input–output curve (not shown; *p* > 0.05).

Application of pirenzepine in the presence of CCh antagonized the CCh-induced decrease of EPSP_max_ (n = 9, *p* > 0.05 *vs.* control and *p* < 0.05 *vs.* CCh; Table 2). Pirenzepine also antagonized the increase of slope factor (n = 9, *p* > 0.05 *vs.* control and *p* < 0.05 *vs.* CCh) and reduced the increase of I_50_ (CCh: 189 ± 39% of control; CCh + Pir:124 ± 12% of control; n = 9, *p* < 0.05 *vs.* control and CCh; Table 2). Application of pirenzepine prior to CCh, however, failed to prevent the CCh-induced decrease of EPSP_max_ (n = 7, *p* < 0.05 *vs.* control or *vs.* pirenzepine alone; Table 2). In fact, CCh added to a pirenzepine-containing ACSF depressed the EPSP_max_ to 76 ± 6% of control (n = 7), indistinguishable from the depression observed with CCh alone (to 69 ± 4% of the control; n = 45; *p* > 0.05). This failure of pirenzepine to prevent a CCh-induced depression, when applied before CCh, is in sharp contrast to the pirenzepine effect on neuronal firing, where pirenzepine is effective irrespective of the sequence of application.

**Table 2:** **Effects of 10 μM CCh, 1 μM pirenzepine, or co-application of CCh and pirenzepine, as indicated, on the maximal amplitude of the field potentials, the maximal amplitude of EPSP (EPSP**_**max**_**), the I**_**50**_**and slope factor of the EPSP input–output curve**

	**Ctl (n = 9)**	**CCh (n = 9)**	**CCh + pir (n = 9)**
**Field max (mV)**	−0.75 ± 0.25	−0.29 ± 0.16 (*)	−0.69 ± 0.24 (+)
**EPSP max (mV)**	24.3 ± 1.5	20.6 ± 2 (*)	23.6 ± 1.7 (+)
**I**_**50**_**(V)**	5.12 ± 0.81	9.23 ± 1.71 (*)	6.22 ± 0.94 (*) (+)
**Slope factor**	1.61 ± 0.59	4.16 ± 0.95 (*)	1.52 ± 0.40 (+)
	**Ctl (n = 7)**	**pir (n = 7)**	**pir + CCh (n = 7)**
**Field max (mV)**	−0.68 ± 0.3	−0.7 ± 0.29	−0.56 ± 0.25 (+)
**EPSP max (mV)**	20.5 ± 5.3	20.3 ± 5.1	16.0 ± 4 (*) (+)
**I**_**50**_**(V)**	8.41 ± 0.75	8.45 ± 0.76	9.37 ± 1.04 (*)
**Slope factor**	1.93 ± 0.54	2.18 ± 0.68	1.95 ± 0.31

The order of two drugs given in the figures indicates the sequence of application (i.e. “CCh + pir” means pirenzepine was added to a CCh containing ACSF and vice versa)

These experiments were performed in STRC

*: *p* < 0.05 *vs.* control, +: *p* < 0.05 *vs.* previous condition

Application of AFDX in the presence of CCh partly antagonized the CCh-induced decrease of the EPSP_max_ (by 52.3 ± 3.1%; n = 8, *p* < 0.05 *vs.* control and CCh; Table 3). AFDX did not antagonize the effect on I_50_ or slope factor (n = 8, *p* > 0.05 *vs.* CCh and *p* < 0.05 *vs.* control; Table 3). Also, application of AFDX before addition of CCh prevented the depressant effect of CCh on EPSP_max_. Indeed, the EPSP_max_ in the presence of AFDX + CCh was not different from that measured in the control condition (n = 7, *p* > 0.05; Table 3). Pre-application of AFDX also prevented the CCh-induced increase of I_50_ (*p* > 0.05 *vs.* control or AFDX alone) but not the increase of slope factor (n = 7, *p* < 0.05 *vs.* control or AFDX alone). To further determine which subtype of mAChR mediates the effects of CCh on EPSP, we applied xanomeline. Xanomeline decreased the EPSP_max_ from 25.6 ± 1.8 mV to 23.2 ± 2.7 mV (n = 12, *p* < 0.05).

**Table 3:** **Effects of 10 μM CCh, 2 μM AFDX, or co-application of CCh and AFDX as indicated on the maximal amplitude of the field potentials, the maximal amplitude of EPSP (EPSP**_**max**_**), the I**_**50**_**and slope factor of the EPSP input–output curve**

	**Ctl (n = 8)**	**CCh (n = 8)**	**CCh + AFDX (n = 8)**
**Field max (mV)**	−0.36 ± 0.09	−0.1 ± 0.04 (*)	−0.26 ± 0.08 (*) (+)
**EPSP max (mV)**	18.9 ± 1.8	10.9 ± 1.6 (*)	15.2 ± 1.7 (*) (+)
**I**_**50**_**(V)**	8.45 ± 0.7	10.9 ± 0.86 (*)	9.65 ± 0.8 (*)
**Slope factor**	2.29 ± 0.27	3.27 ± 0.48 (*)	2.70 ± 0.33 (*)
	**Ctl (n = 7)**	**AFDX (n = 7)**	**AFDX + CCh (n = 7)**
**Field max (mV)**	−0.79 ± 0.17	−0.99 ± 0.24	−0.67 ± 0.23
**EPSP max (mV)**	22.2 ± 2.4	23.9 ± 1.8 (*)	23 ± 1.8
**I**_**50**_**(V)**	7.42 ± 1.11	7.22 ± 1.44	8.94 ± 2.37
**Slope factor**	1.82 ± 0.67	2.05 ± 0.71	3.07 ± 0.75 (*) (+)

The order of two drugs given in the figures indicates the sequence of application (i.e. “CCh + AFDX” means AFDX was added to a CCh containing ACSF and vice versa)

These experiments were performed in STRC

*: *p* < 0.05 *vs.* control, +: *p* < 0.05 *vs.* previous condition

#### CCh-induced depression of synaptic responses is not mimicked by M-current blockade

**Figure 6:**
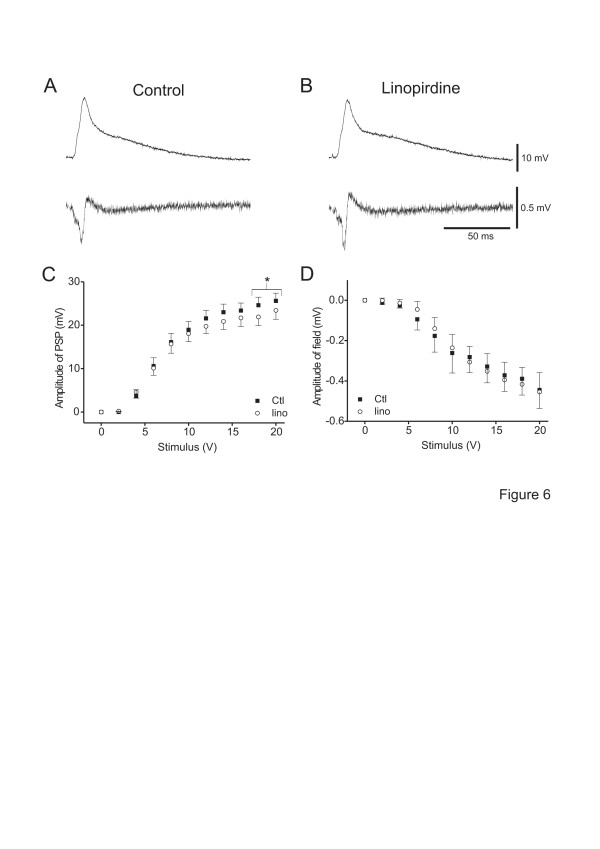
**CCh-induced depression of synaptic transmission is not due to blockade of M-current.** A, B. Voltage traces (top traces) and field potentials (bottom traces) of a neocortical neurone in control condition (A) and in the presence of linopirdine (10 μM) (B; stimulus intensity 100 μs, 20 V). Note that linopirdine did not alter synaptic responses. C, D. Plots of average EPSP amplitudes (C; n = 12) and field potentials (D; n = 12) *vs.* stimulus intensity in control and in the presence of linopirdine (lino) as indicated. Note that linopirdine application decreased the amplitude of EPSP only at the highest stimulus intensities. These experiments were performed in STRC. *: *p* < 0.05

Linopirdine had no effect on the amplitude of the field potentials (Figure 6A, B, bottom traces, 6D and only marginally decreased (< 10%) the amplitude of EPSP (Figure 6A, B, top traces). This effect was only present at higher stimulus intensities (n = 12, *p* < 0.05; Figure 6C). Linopirdine had no effect on the slope factor but slightly reduced I_50_ (from 6.9 ± 0.6 V to 6.4 ± 0.6 V; n = 12, *p* < 0.05). Retigabine (10 μM) had no effect on the maximal amplitude of the field potentials (control: -0.54 ± 0.13 V, retigabine: -0.53 ± 0.13 V), on EPSP_max_ (control: 30.8 ± 3.4 V, retigabine: 30.5 ± 4.0 V), or the slope factor (control: 1.01 ± 0.26 V, retigabine: 1.12 ± 0.24 V) (n = 8, *p* > 0.05 for all). However, retigabine increased I_50_ (from 11.3 ± 0.8 V to 13.2 ± 0.9 V; n = 8, *p* < 0.05). These data suggest that CCh–induced depression of maximal field potentials and EPSP_max_ are mediated by mAChRs not coupled to K_V_7 channels.

## Discussion

The key findings of this study are: (i) activation of M_1_ mAChR increased the neuronal AP firing via blockade of M-current, (ii) activation of M_2_ mAChR increased the paired-pulse ratio of field potentials and decreased both components of GABAergic inhibition, and (iii) activation of M_4_ mAChR depressed glutamatergic transmission presumably via activation of presynaptic receptors.

### Methodological aspects

The previous studies investigating the mAChRs in slice preparations used either sharp microelectrode recordings [20,30] or whole cell patch clamp recordings [22,23,31,32]. The latter disturb the intracellular milieu and a massive rundown of M-current (modulated by mAChRs) was observed [33], precluding a reliable pharmacology. Intracellular recordings in cortical slices with sharp microelectrodes hence present a valuable method to quantify the effects at native mAChRs in slices.

However, reports in the neocortex focussed on the role of mAChRs or M-current either on the neuronal firing [20,30,32], on the GABAergic transmission [23,31] or on the glutamatergic transmission [22,34]. Only few studies in cortex e.g. [21] or in hippocampus e.g. [35] investigated the role of mAChRs on both neuronal firing and synaptic transmission. The comparison of data obtained in different preparations using different experimental conditions is problematic, though. Therefore, in the present study we investigated the 3 aspects, namely change of firing, depression of EPSPs and of IPSPs, in the same preparation under identical experimental conditions.

Although some authors used different concentrations of mAChRs agonists/antagonists, only one or few stimulus intensities e.g. [22] or current injections e.g. [35] were used. Therefore, the results were mostly qualitative from a physiological point of view, precluding quantitative comparisons of different agonists/antagonists.

### Effect of mAChR activation on neuronal excitability

The consistent increase in the slope of neuronal firing by CCh in the somatosensory cortex is similar to the hippocampus [35] and the nucleus gracilis [36]. However, our determination of slope and intercept provides a more quantitative analysis of changes of firing compared to previous observations [35,36]. In particular, these authors compared the neuronal firing in response to a single current intensity before and during substance application. The interpretation of such data would be complicated by concomitant alterations of membrane potential and/or neuronal input resistance.

In any case, alterations of firing are mediated by mAChRs, as evidenced by the antagonism by atropine. Moreover, the antagonism by pirenzepine (M_1_/M_4_ mAChRs antagonist) but not by AFDX (M_2_/M_4_ mAChRs antagonist) further suggests an effect via M_1_ mAChR, similar to the M_1_ mAChR-mediated repetitive spontaneous neuronal firing observed in rat prefrontal cortex [21]. Our conclusion is in line with previous data indicating that ACh application increases the neuronal firing of neocortical pyramidal neurones in mice lacking M_3_ or M_5_ mAChRs but not in mice lacking M_1_ mAChR, confirming further an involvement of the M_1_ mAChR in this effect [37].

The cellular mechanisms underlying the CCh-induced increase of AP firing can be inferred from the effect of linopirdine which is an established blocker of K_V_7 channels [25]. Linopirdine increased the neuronal firing as in hippocampus [25] and entorhinal cortex [32]. Together, these data indicate a M_1_ mAChR mediated depression of M-current [38] as the underlying mechanism of CCh-induced firing increase.

### Effect of mAChR activation on GABAergic transmission

We studied the mAChR mediated effect on neurotransmitter release by using the paired-pulse protocol with field potentials. CCh increased the PPR for interpulse intervals ranging from 10 to 400 ms. This suggests that the CCh effect involves both GABA_A_ and GABA_B_ receptor-mediated effects. The CCh-induced increase of the PPR is most likely due to mAChR-mediated depression of GABA release [23], and evoked IPSCs [31], during the first stimulus. Normally, GABA would attenuate the response to a second stimulus by decreasing release via presynaptic GABA_B_ receptors [39,40]. Hence a mAChR-mediated depression of GABA release might contribute to the altered paired pulse properties of synaptic responses.

The involvement of M_2_ mAChR in the CCh-induced depression of GABA release underlying increase of PPR can be inferred from the antagonism by AFDX (M_2_/M_4_ mAChRs antagonist) and lack of antagonism by pirenzepine (M_1_/M_4_ mAChRs antagonist). Similar to the pharmacology at M_1_ mAChR, the effects of atropine and AFDX on this M_2_ mAChR-mediated increase of PPR were identical whether the antagonist was applied in the presence of, or before, CCh.

Further evidence was obtained by evaluating the alterations in inhibitory conductance mediated by GABA_A_ and GABA_B_ receptors. Firstly, the similarity in the depression of both g_IPSP-A_ and g_IPSP-B_, indicates a common denominator for both effects i.e. depression of GABA release. The effects of the selected compounds revealed which mAChR is involved in this depression of GABA release. The CCh effects were considerably antagonized by the M_2_/M_4_ mAChRs antagonist AFDX but slightly by the M_1_/M_4_ mAChRs antagonist pirenzepine. Moreover, the M_1_/M_4_ mAChRs agonist xanomeline had no effects on g_IPSP-A_ or g_IPSP-B_, yet subsequent addition of CCh reduced g_IPSP-A_ and g_IPSP_. This observation strongly suggests that neither M_1_ nor M_4_ mAChRs are involved in the modulation of GABA release. Together these data support our view that M_2_ mAChR are involved in the modulation of GABA release.

This view is also supported by histological data. M_2_ mAChRs are predominantly localized at presynaptic axons of GABAergic neurones and are associated with asymmetric as well as symmetric cortical synapses [16-18,23], suggesting that M_2_ mAChR can modulate GABA release in the neocortex. In addition, M_1_ mAChRs are mainly expressed on cortical pyramidal neurones rather than by GABAergic neurones [41].

ACh reduces the GABA release in neocortex by two separate mechanisms i) by decreasing Ca^2+^ currents via activation of a M_2_ mAChR/PI3K/Ca^2+^-independent PKC pathway and to a smaller extent by activation of a M_1_ mAChR/PLC/Ca^2+^-dependent PKC pathway [23]. This combination might explain the relatively large contribution of the M_2_ mAChR and the absence of detectable effects of the M_1_ mAChR in the CCh-induced increase of PPR observed here.

### Effect of mAChR activation on glutamatergic transmission

Our observation of CCh decreasing the amplitudes of evoked synaptic potentials is in line with a wealth of evidence from various areas of the rodent brain [22,34-36]. Both field potentials and intracellularly recorded EPSPs were depressed by CCh to a similar degree. Considering the marginal changes in E_m_, this decrease cannot be accounted for by a membrane depolarization, confirming results obtained in entorhinal cortex [34]. These authors reported that the depression of synaptic transmission persisted when neurones were returned to their initial resting potential. However, previous observations in hippocampus e.g. [35] or neocortex e.g. [21,22] compared the synaptic response following a single stimulus intensity before and during substance application. Our determination of I_50_, EPSP_max_ and slope factor provide a more quantitative analysis of changes of synaptic transmission, corroborating and extending previous findings. In particular, the consistent effects over a wide range of stimulus intensities exclude the possibility of threshold phenomena.

Our data indicate that this CCh-induced depression of EPSP is 1) fully prevented when CCh is applied in the presence of atropine or AFDX and 2) only partially prevented when CCh was applied in the presence of pirenzepine. This corroborates and extends previous data from layer V of the somatosensory cortex [22]. Unlike these authors, applying CCh in the presence of antagonists, we also applied the mAChRs antagonists in the presence of CCh, revealing marked differences with different sequence of application. The CCh-mediated depression of EPSP was fully reversed when pirenzepine or atropine was added in the presence of CCh, i.e. pirenzepine is more effective when applied in the presence of the agonist. AFDX, however, only partially reversed this depression, i.e. is less effective when applied in the presence of the agonist. This peculiarity may contribute to the discrepancies between previous reports. Usually, the authors have either applied the antagonist in the presence of agonist or vice versa, the agonist in the presence of antagonists. To the best of our knowledge, the two complementary protocols had not been used in the same study so far e.g. either addition or pre-application was used, see [22,42].

We propose that pirenzepine, a M_1_/M_4_ prefering mAChRs antagonist [43], affects the EPSP amplitude via M_4_ mAChR for the following reasons:

1) The increase of neuronal firing by CCh was prevented and reversed by pirenzepine. However, the decrease of EPSP_max_ by CCh was reversed but not prevented by pirenzepine. This difference may suggest that the pirenzepine-sensitive effects on EPSP_max_ and on slope of neuronal firing are mediated by two distinct mAChRs, i.e. the pirenzepine-sensitive effect on EPSP_max_ is not mediated by M_1_ mAChR.

2) Only M_2_ and M_4_ mAChRs (but not M_1_, M_3_ and M_5_ mAChRs) are coupled to G-proteins G_i_/G_o_ family involved in the inhibition of neurotransmitter release [7,44,45]. In addition, recent evidence indicates that M_4_ mAChR is the major mAChR subtype responsible for direct cholinergic modulation of EPSP [46]. Therefore, the pirenzepine effect on CCh-induced EPSP depression is probably due to an action of pirenzepine on M_4_ rather than M_1_ mAChRs [43]. This view is supported by immunohistochemical data [17], showing that cortical M_1_ mAChRs are located exclusively at postsynaptic sites.

3) The effects of atropine and AFDX on the M_2_ mAChR-mediated increase of PPR were identical regardless of the sequence of application. However, the effects of pirenzepine and AFDX on the CCh-induced decrease of EPSP were different whether this antagonist is applied in the presence or before the CCh application. This difference may suggest that the CCh-induced decrease of EPSP_max_ and increase of PPR are mediated by two distinct mAChRs, i.e. the CCh-induced decrease of EPSP_max_ is not mediated by M_2_ mAChR. Moreover, the increase of PPR by CCh (mediated by M_2_ mAChR) was not prevented or reversed by pirenzepine. This suggests that pirenzepine is not active at M_2_ mAChR in our conditions.

4) AFDX applied before CCh or pirenzepine applied in the presence of CCh can both fully prevent and reverse, respectively, the CCh-induced depression of EPSP_max_. The similar potency of pirenzepine and AFDX on CCh-induced EPSP_max_ depression therefore suggests an involvement of M_4_ mAChR as the most parsimonious explanation.

### Cholinergic transmission and cognition

Ample evidence suggests that mAChR-mediated signalling in the cortex is critical for learning and memory, yet the mechanisms of ACh facilitating cognitive processes remained elusive. The effects reported here, namely the depression of EPSP and the increased neuronal firing mediated by different mAChRs would provide a very interesting system for “attention” at the cellular level. The M_4_ mAChR-mediated depression of EPSP reduces the noise of ongoing synaptic activity while the M_1_ mAChR-mediated increase of neuronal firing augments the neuronal response to a given synaptic input, i.e. these two effects would improve the signal to noise ratio. Moreover, the shallower input–output curve of synaptic responses induced by M_4_ mAChR observed here extends the subthreshold range for temporal summation, which is augmented by a CCh-induced reduction of Kir-type potassium current [21]. In addition, the cholinergic depression of GABA release corresponds to a depression of inhibition by higher frequencies of stimulation [40] implicated in long-term potentiation [47]. Together, these data corroborate and extend a theoretical framework proposed by Hasselmo and McGaughy [48].

## Conclusions

Our data provide evidence that the triade of cellular CCh effects are mediated by three subtypes of mAChR, i) M_1_ mAChR-mediated increase of firing, ii) M_2_ mAChR-mediated depression of GABA release and iii) M_4_ mAChR-mediated depression of glutamatergic synaptic responses. Activation of these effects by physiologically released ACh provides an interesting combination of neuronal attention and facilitation of LTP. In addition, the three parameters delineated here may present useful tools to test new M_1_, M_2_ or M_4_ mAChRs modulators at native receptors in the human neocortex from epilepsy surgery tissues.

## Methods

### Tissue handling and preparation

Coronal slices containing the sensorimotor cortex were made from male Wistar rats (age: 30-42 d). Experiments were approved by the Berlin health protection agency (T 026/96). Rats were deeply anesthetized with ether, decapitated and a block of brain containing the sensorimotor cortex was quickly removed. The tissue was cut into slices (400 μm) with a vibratome (HM 650 V, MICROM International, Walldorf, Germany) in artificial cerebrospinal fluid (ACSF, 5 ± 1°C). The slices were stored submerged at room temperature in continuously oxygenated (carbogen: 95% O_2_-5% CO_2_) ACSF. Slices were allowed to recover for at least 1 hour before recordings commenced. Individual slices were transferred to the recording chambers and given 30 min to adapt.

### Solutions and substances

ACSF contained (in mM) 124 NaCl, 5 KCl, 2 MgSO_4_, 2 CaCl_2_, 1.25 NaH_2_PO_4_, 26 NaHCO_3_ and 10 glucose (pH 7.4, after equilibration with carbogen).

Atropine (Sigma, Taufkirchen, Germany), carbamylcholine-chloride (carbachol, CCh; Gift from GlaxoSmithKline), linopirdine dihydrochloride (Tocris, Bristol, UK) and pirenzepine (Sigma) were dissolved in H_2_O. AF-DX 116 (abbreviated AFDX, Tocris), retigabine and xanomeline (both gift from GlaxoSmithKline) were dissolved in DMSO. The final concentration of DMSO was below 0.1%. Stock solutions of each drug were prepared and stored at −20°C until use.

The following mAChRs related compounds were used: pirenzepine (M_1_/M_4_ mAChRs antagonist), AFDX 116 (M_2_/M_4_ mAChRs antagonist), xanomeline (M_1_/M_4_ mAChRs agonist) and atropine (broad spectrum mAChRs antagonist) [24,43,49]. We refrained from using mamba toxin 7, a proposed selective blocker of M_1_ mAChR, because the application via perfusion would be prohibitively expensive and the stability under our recording conditions (oxygen, 32°C) is uncertain [communicated by a manufacturer]. We chose established concentrations of agonist and antagonist based on a literature survey and a few pilot experiments. A concentration of 10 μM CCh and 1 μM atropine is established in slices [21,22], and at 1 μM, atropine does not interfere with nicotinic receptors [50]. The concentrations of pirenzepine (1 μM) and AFDX (2 μM) were chosen from binding experiments [43,49] and are effective on the CCh-induced depression of PSP in neocortex or hippocampus slices for example [22,51]. Pirenzepine at 1 μM should have a maximal effect at M_1_ and M_4_ mAChRs but not at M_2_, M_3_ and M_5_ mAChRs. AFDX at 2 μM should have a maximal effect at M_2_ and M_4_ mAChRs but not at M_1_, M_3_ and M_5_ mAChR [49].

From the above considerations, the following predictions can be made with a minimum of compounds: 1) a M_1_ mAChR-mediated effect should be antagonized by pirenzepine but not by AFDX, 2) a M_2_ mAChR-mediated effect should be antagonized by AFDX but not by pirenzepine and 3) a M_4_ mAChR-mediated effect should be antagonized by either pirenzepine or AFDX.

To reveal more subtle pharmacological differences of the mAChRs, i.e. effects of activation state, we applied antagonists either before or during the CCh application.

### Electrophysiological recordings

#### Recording chambers

Experiments were carried out either in an interface-type recording chamber (ITRC) or in a submersion-type recording chamber (STRC). The ITRC enables maximal oxygen supply and improves recordings of evoked field potentials and was therefore chosen for the paired-pulse protocol. The STRC provides more stable conditions and allows a faster equilibration of drugs in the slices [28]. Therefore, STRC was chosen for intracellular recordings.

In ITRC, slices were perfused with oxygenated ACSF (1.5-2 ml/min; 34 ± 1°C) and the atmosphere was maintained by a continuous flow of pre-warmed and humidified carbogen. In STRC experiments slices were held between two nylon grids and perfused with a higher flow rate of oxygenated ACSF (4–5 ml/min; 31 ± 1°C).

All measurements were made at least 30 min (ITRC) or 15 min (STRC) after drug application to ascertain steady-state of applied drugs.

#### Electrodes

For extracellular recordings, filamented borosilicate capillaries (Hilgenberg, Malsfeld, Germany) were pulled on a DMZ universal puller (Zeitz, München, Germany). The pipette resistances were 2–8 MΩ when filled with ACSF. Sharp microelectrodes for intracellular recordings were pulled on a Flaming/Brown P87 Puller (Sutter Instruments, Novato, U.S.A.). When filled with 1 M potassium acetate (pH 7.2, containing also 1 mM KCl and 5 mM EGTA) the electrodes had resistances between 70 and 120 MΩ.

#### Extra- and intracellular recordings

The electrodes were positioned under visual control in neocortical layers II/III. Signals obtained with extracellular recordings were fed to a high-impedance preamplifier (EXT-01 C, npi electronic, Tamm, Germany) and processed through second-stage amplifiers with filtering capabilities (DPA 2 F; npi electronic, Tamm, Germany). The signals obtained with intracellular recordings were fed to an appropriate amplifier (SEC-05 L, npi electronic, Tamm, Germany).

We evaluated the current voltage relationship from families of current injections (between −0.5 nA and +1.5 nA, increment 0.05 nA, duration 600 ms), to estimate the neuronal input resistance and the firing behaviour. Linear regression of the number of APs *vs.* injected current in the initial linear range was used to calculate the slope and the intercept. The former parameter provides a quantitative index of AP firing behaviour in a given condition. The latter represents the current just below the rheobase and was termed sub-rheobase.

Synaptic responses were elicited by electrical stimuli (0–20 V, increment 2 V, 100 μs duration at 0.1 Hz, in triplicate) applied via a bipolar tungsten electrode placed in the deeper cortical layers (V/VI). The peak amplitude of the initial component represents the excitatory postsynaptic potential and will be referred to as EPSP, although it might be slightly affected by the inhibitory postsynaptic potential (IPSP) with a slightly later time to peak (see Figure 4A). The input–output curves of EPSP were fitted by the Boltzmann equation:

(1) $$\text{Y}=\left({\text{A}}_{1}–{\text{A}}_{2}\right)/\left(1+{\text{e}}^{\left(\text{x }-\text{ xo}\right)/\text{dx}}\right)+{\text{A}}_{2}$$

yielding the maximal EPSP amplitude (A_2_, EPSP_max_), the half maximal stimulus (x_0_, I_50_) and the slope factor (dx).

Synaptic conductance mediated by GABA_A_ and GABA_B_ receptors was estimated similar to previous methods [40,52]. In brief, linear regressions of the current voltage relation (usually between −0.3 and +0.3 nA) was carried out before the stimulus and at the apparent peak of the early and late inhibitory synaptic responses (near 20 ms and 150 ms poststimulus). Assuming synaptic conductances parallel to “resting” membrane conductance, the subtraction of resting membrane conductance from the total conductance at the peak of GABA_A_ and GABA_B_ receptor-mediated events yields an estimate for the two synaptic responses see [40].

To evaluate the effects of mAChR agonists/antagonists on neurotransmitter release supposedly via presynaptic sites, we recorded the field potentials during a paired-pulse protocol in an ITRC. The ratio of amplitude of the second field potential in relation to the first in response to identical stimulations (100 μs, 1 mA) was calculated for different interstimulus intervals (10 ms-1 s) and was termed paired-pulse ratio (PPR). An intensity of 1 mA was chosen for this protocol since it induced a maximal response with pronounced paired-pulse depression in all slices tested. In addition, we performed input–output curves, as in STRC. In ITRC, field potentials were elicited in cortical layers II/III by electrical stimuli (0-1 mA, increment 0.1 mA, 100 μs duration at 0.1 Hz, in triplicate) delivered via a bipolar tungsten electrode placed in cortical layers V/VI.

### Data acquisition and analysis

Recorded signals were digitized on-line (10 kHz) with a PC based system (Digidata 1200 and Clampex 9.3 or Digidata 1440A and Clampex 10.1 software, Molecular Devices, Sunnyvale CA, USA), stored on hard disk and analysed off-line (Clampfit 10.1).

Our intracellular recordings were from “regular firing” neurones, as opposed to “fast spiking” or “burst firing” neurones [53].

Data are presented as mean ± s.e.m., n indicates the number of cells (only one cell per slice was recorded). For comparisons, we used paired or unpaired Student’s *t*-test. Differences with *p* < 0.05 were considered significant.

## Abbreviations

ACh, Acetylcholine; ACSF, Artificial cerebrospinal fluid; AFDX, AF-DX 116; CCh, Carbamylcholine-chloride (carbachol); Em, Resting membrane potential; EPSP, Excitatory postsynaptic potentials; IPSP, Inhibitory postsynaptic potentials; ITRC, Interface-type recording chamber; LTP, Long term potentiation; mAChR, Muscarinic acetylcholine receptor; PPR, Paired-pulse ratio; Rm, Input resistance; STRC, Submersion-type recording chamber.

## Authors’ contributions

S.G. and R.A.D. did the experiments, data analysis and writing of the manuscript. S.W. assisted during the experiments and provided helpful comments on the manuscript. G.A.J. C.H.D. and J.M.W. initiated these experiments and commented on a preliminary version of the manuscript.

## References

[B1] Wess J Muscarinic acetylcholine receptor knockout mice: novel phenotypes and clinical implications Annu Rev Pharmacol Toxicol 2004 44 423 450 10.1146/annurev.pharmtox.44.101802.121622 14744253

[B2] Freedman R Adler LE Bickford P Byerley W Coon H Cullum CM Griffith JM Harris JG Leonard S Miller C Schizophrenia and nicotinic receptors Harv Rev Psychiatry 1994 2 4 179 192 10.3109/10673229409017136 9384901

[B3] Harbaugh RE Roberts DW Coombs DW Saunders RL Reeder TM Preliminary report: intracranial cholinergic drug infusion in patients with Alzheimer’s disease Neurosurgery 1984 15 4 514 518 10.1227/00006123-198410000-00007 6149490

[B4] Minzenberg MJ Poole JH Benton C Vinogradov S Association of anticholinergic load with impairment of complex attention and memory in schizophrenia Am J Psychiatry 2004 161 1 116 124 10.1176/appi.ajp.161.1.116 14702259

[B5] Fisher A Cholinergic treatments with emphasis on m1 muscarinic agonists as potential disease-modifying agents for Alzheimer’s disease Neurotherapeutics 2008 5 3 433 442 10.1016/j.nurt.2008.05.002 18625455 PMC5084245

[B6] Pepeu G Giovannini MG Cholinesterase inhibitors and beyond Curr Alzheimer Res 2009 6 2 86 96 10.2174/156720509787602861 19355843

[B7] Caulfield MP Birdsall NJM International union of pharmacology. XVII. Classification of muscarinic acetylcholine receptors Pharmacol Rev 1998 50 2 279 290 9647869

[B8] Peralta EG Ashkenazi A Winslow JW Smith DH Ramachandran J Capon DJ Distinct primary structures, ligand-binding properties and tissue-specific expression of 4 human muscarinic acetylcholine-receptors EMBO J 1987 6 13 3923 3929 3443095 10.1002/j.1460-2075.1987.tb02733.x PMC553870

[B9] Delmas P Brown DA Pathways modulating neural KCNQ/M (Kv7) potassium channels Nat Rev Neurosci 2005 6 11 850 862 16261179 10.1038/nrn1785

[B10] Langmead CJ Watson J Reavill C Muscarinic acetylcholine receptors as CNS drug targets Pharmacol Ther 2008 117 2 232 243 10.1016/j.pharmthera.2007.09.009 18082893

[B11] Clader JW Wang Y Muscarinic receptor agonists and antagonists in the treatment of Alzheimer’s disease Curr Pharm Des 2005 11 26 3353 3361 10.2174/138161205774370762 16250841

[B12] Olianas MC Dedoni S Ambu R Onali P Agonist activity of N-desmethylclozapine at delta-opioid receptors of human frontal cortex Eur J Pharmacol 2009 607 1–3 96 101 19239909 10.1016/j.ejphar.2009.02.025

[B13] Fleming JJ England PM Developing a complete pharmacology for AMPA receptors: a perspective on subtype-selective ligands Bioorg Med Chem 2010 18 4 1381 1387 10.1016/j.bmc.2009.12.072 20096591

[B14] Thomas DR Dada A Jones GA Deisz RA Gigout S Langmead CJ Werry TD Hendry N Hagan JJ Davies CH N-desmethylclozapine (NDMC) is an antagonist at the human native muscarinic M1 receptor Neuropharmacology 2010 58 8 1206 1214 10.1016/j.neuropharm.2010.02.017 20206188

[B15] Levey AI Kitt CA Simonds WF Price DL Brann MR Identification and localization of muscarinic acetylcholine receptor proteins in brain with subtype-specific antibodies J Neurosci 1991 11 10 3218 3226 1941081 10.1523/JNEUROSCI.11-10-03218.1991 PMC6575445

[B16] Mrzljak L Levey AI Belcher S Goldman-Rakic PS Localization of the m2 muscarinic acetylcholine receptor protein and mRNA in cortical neurons of the normal and cholinergically deafferented rhesus monkey J Comp Neurol 1998 390 1 112 132 10.1002/(SICI)1096-9861(19980105)390:1<112::AID-CNE10>3.0.CO;2-Z 9456180

[B17] Mrzljak L Levey AI Goldman-Rakic PS Association of m1 and m2 muscarinic receptor proteins with asymmetric synapses in the primate cerebral cortex: morphological evidence for cholinergic modulation of excitatory neurotransmission Proc Natl Acad Sci USA 1993 90 11 5194 5198 10.1073/pnas.90.11.5194 8389473 PMC46682

[B18] Disney AA Domakonda KV Aoki C Differential expression of muscarinic acetylcholine receptors across excitatory and inhibitory cells in visual cortical areas V1 and V2 of the macaque monkey J Comp Neurol 2006 499 1 49 63 10.1002/cne.21096 16958109

[B19] Krnjevic K Pumain R Renaud L The mechanism of excitation by acetylcholine in the cerebral cortex J Physiol (Lond) 1971 215 1 247 268 5579661 10.1113/jphysiol.1971.sp009467 PMC1331876

[B20] McCormick DA Williamson A Convergence and divergence of neurotransmitter action in human cerebral cortex Proc Natl Acad Sci USA 1989 86 20 8098 8102 10.1073/pnas.86.20.8098 2573061 PMC298222

[B21] Carr DB Surmeier DJ M1 muscarinic receptor modulation of Kir2 channels enhances temporal summation of excitatory synaptic potentials in prefrontal cortex pyramidal neurons J Neurophysiol 2007 97 5 3432 3438 10.1152/jn.00828.2006 17376848

[B22] Levy RB Reyes AD Aoki C Nicotinic and muscarinic reduction of unitary excitatory postsynaptic potentials in sensory cortex; Dual intracellular recording in vitro J Neurophysiol 2006 95 4 2155 2166 16421199 10.1152/jn.00603.2005 PMC1409808

[B23] Salgado H Bellay T Nichols JA Bose M Martinolich L Perrotti L Atzori M Muscarinic M2 and M1 receptors reduce GABA release by Ca2+ channel modulation through activation of PI3K/Ca2+-independent and PLC/Ca2+-dependent PKC J Neurophysiol 2007 98 2 952 965 10.1152/jn.00060.2007 17581851

[B24] Shannon HE Rasmussen K Bymaster FP Hart JC Peters SC Swedberg MDB Jeppesen L Sheardown MJ Sauerberg P Fink-Jensen A Xanomeline, an M1/M4 preferring muscarinic cholinergic receptor agonist, produces antipsychotic-like activity in rats and mice Schizophrenia Research 2000 42 3 249 259 10.1016/S0920-9964(99)00138-3 10785583

[B25] Aiken SP Lampe BJ Murphy PA Brown BS Reduction of spike frequency adaptation and blockade of M-current in rat CA1 pyramidal neurones by linopirdine (DuP 996), a neurotransmitter release enhancer Br J Pharmacol 1995 115 7 1163 1168 10.1111/j.1476-5381.1995.tb15019.x 7582539 PMC1908770

[B26] Rundfeldt C The new anticonvulsant retigabine (D-23129) acts as an opener of K+ channels in neuronal cells Eur J Pharmacol 1997 336 2–3 243 249 9384239 10.1016/s0014-2999(97)01249-1

[B27] Luhmann HJ Mittmann T Vanluijtelaar G Heinemann U Impairment of intracortical GABAergic inhibition in a rat model of absence epilepsy Epilepsy Res 1995 22 1 43 51 10.1016/0920-1211(95)00032-6 8565966

[B28] Reid KH Edmonds HL Schurr A Tseng MT West CA Pitfalls in the use of brain slices Prog Neurobiol 1988 31 1 1 18 10.1016/0301-0082(88)90020-2 3287453

[B29] Thompson SM Deisz RA Prince DA Relative contributions of passive equilibrium and active transport to the distribution of chloride in mammalian cortical neurons J Neurophysiol 1988 60 1 105 24 3404212 10.1152/jn.1988.60.1.105

[B30] McCormick DA Prince DA Two types of muscarinic response to acetylcholine in mammalian cortical neurons Proc Natl Acad Sci USA 1985 82 18 6344 6348 10.1073/pnas.82.18.6344 3862134 PMC391050

[B31] Xiao Z Deng PY Yang C Lei S Modulation of GABAergic transmission by muscarinic receptors in the entorhinal cortex of juvenile rats J Neurophysiol 2009 102 2 659 669 10.1152/jn.00226.2009 19494196 PMC2724340

[B32] Yoshida M Alonso A Cell-type specific modulation of intrinsic firing properties and subthreshold membrane oscillations by the M(Kv7)-current in neurons of the entorhinal cortex J Neurophysiol 2007 98 5 2779 2794 10.1152/jn.00033.2007 17728392

[B33] Shen W Hamilton SE Nathanson NM Surmeier DJ Cholinergic suppression of KCNQ channel currents enhances excitability of striatal medium spiny neurons J Neurosci 2005 25 32 7449 7458 10.1523/JNEUROSCI.1381-05.2005 16093396 PMC6725301

[B34] Hamam BN Sinai M Poirier G Chapman CA Cholinergic suppression of excitatory synaptic responses in layer II of the medial entorhinal cortex Hippocampus 2007 17 2 103 113 10.1002/hipo.20249 17146776

[B35] Sheridan RD Sutor B Presynaptic M1 muscarinic cholinoceptors mediate inhibition of excitatory synaptic transmission in the hippocampus in vitro Neurosci Lett 1990 108 3 273 278 10.1016/0304-3940(90)90653-Q 2304648

[B36] De Fernandez Sevilla D Rodrigo-Angulo M Nunez A Buno W Cholinergic modulation of synaptic transmission and postsynaptic excitability in the rat gracilis dorsal column nucleus J Neurosci 2006 26 15 4015 4025 10.1523/JNEUROSCI.5489-05.2006 16611818 PMC6673877

[B37] Gulledge AT Bucci DJ Zhang SS Matsui M Yeh HH M1 receptors mediate cholinergic modulation of excitability in neocortical pyramidal neurons J Neurosci 2009 29 31 9888 9902 10.1523/JNEUROSCI.1366-09.2009 19657040 PMC2745329

[B38] Hamilton SE Loose MD Qi M Levey AI Hille B McKnight GS Idzerda RL Nathanson NM Disruption of the m1 receptor gene ablates muscarinic receptor-dependent M current regulation and seizure activity in mice Proc Natl Acad Sci U S A 1997 94 24 13311 13316 10.1073/pnas.94.24.13311 9371842 PMC24305

[B39] Deisz RA The GABAB receptor antagonist CGP 55845A reduces presynaptic GABAB actions in neocortical neurons of the rat in vitro Neuroscience 1999 93 4 1241 1249 10.1016/S0306-4522(99)00203-1 10501448

[B40] Deisz RA Prince DA Frequency-dependent depression of inhibition in guinea-pig neocortex in vitro by GABAB receptor feed-back on GABA release J Physiol (Lond) 1989 412 513 541 2557431 10.1113/jphysiol.1989.sp017629 PMC1190589

[B41] Yamasaki M Matsui M Watanabe M Preferential localization of muscarinic M1 receptor on dendritic shaft and spine of cortical pyramidal cells and its anatomical evidence for volume transmission J Neurosci 2010 30 12 4408 4418 10.1523/JNEUROSCI.5719-09.2010 20335477 PMC6634497

[B42] Richter M Schilling T Muller W Muscarinic control of intracortical connections to layer II in rat entorhinal cortex slice Neurosci Lett 1999 273 3 200 202 10.1016/S0304-3940(99)00643-6 10515193

[B43] Moriya H Takagi Y Nakanishi T Hayashi M Tani T Hirotsu I Affinity profiles of various muscarinic antagonists for cloned human muscarinic acetylcholine receptor (mAChR) subtypes and mAChRs in rat heart and submandibular gland Life Sci 1999 64 25 2351 2358 10.1016/S0024-3205(99)00188-5 10374898

[B44] Caulfield MP Muscarinic receptors - Characterization, coupling and function Pharmacol Ther 1993 58 3 319 379 10.1016/0163-7258(93)90027-B 7504306

[B45] Lanzafame AA Christopoulos A Mitchelson F Cellular signaling mechanisms for muscarinic acetylcholine receptors Recept Channels 2003 9 4 241 260 10.1080/10606820308263 12893537

[B46] Dasari S Gulledge AT M1 and M4 receptors modulate hippocampal pyramidal neurons J Neurophysiol 2011 105 2 779 792 10.1152/jn.00686.2010 21160001 PMC3059175

[B47] Davies CH Starkey SJ Pozza MF Collingridge GL GABA autoreceptors regulate the induction of LTP Nature 1991 349 6310 609 611 10.1038/349609a0 1847993

[B48] Hasselmo ME McGaughy J High acetylcholine levels set circuit dynamics for attention and encoding and low acetylcholine levels set dynamics for consolidation Prog Brain Res 2004 145 207 231 14650918 10.1016/S0079-6123(03)45015-2

[B49] Buckley NJ Bonner TI Buckley CM Brann MR Antagonist binding properties of five cloned muscarinic receptors expressed in CHO-K1 cells Mol Pharmacol 1989 35 4 469 476 2704370

[B50] Alkondon M Pereira EFR Almeida LEF Randall WR Albuquerque EX Nicotine at concentrations found in cigarette smokers activates and desensitizes nicotinic acetylcholine receptors in CA1 interneurons of rat hippocampus Neuropharmacology 2000 39 13 2726 2739 10.1016/S0028-3908(00)00156-8 11044743

[B51] Dutar P Nicoll RA Classification of muscarinic responses in hippocampus in terms of receptor subtypes and second-messenger systems: electrophysiological studies in vitro J Neurosci 1988 8 11 4214 4224 3141594 10.1523/JNEUROSCI.08-11-04214.1988 PMC6569473

[B52] Teichgraber LA Lehmann TN Meencke HJ Weiss T Nitsch R Deisz RA Impaired function of GABAB receptors in tissues from pharmacoresistant epilepsy patients Epilepsia 2009 50 7 1697 1716 10.1111/j.1528-1167.2009.02094.x 19453710

[B53] Connors BW Gutnick MJ Intrinsic firing patterns of diverse neocortical neurons Trends Neurosci 1990 13 3 99 104 10.1016/0166-2236(90)90185-D 1691879

